# 675. The Fidaxomicin Cascade: Analysis of Success Rates and Barriers of Fidaxomicin Discharge Prescriptions for Inpatients Diagnosed with Clostridiodes Difficile

**DOI:** 10.1093/ofid/ofad500.737

**Published:** 2023-11-27

**Authors:** Ellen Cook, Jigar Mehta, Alexis L Blecher, Matthew S Lee

**Affiliations:** Beth Israel Deaconess Medical Center, Boston, Massachusetts; Beth Israel Deaconess Medical Center, Boston, Massachusetts; Beth Israel Deaconess Medical Center, Boston, Massachusetts; Beth Israel Deaconess Medical Center, Boston, Massachusetts

## Abstract

**Background:**

Current IDSA guidelines prefer Fidaxomicin for initial and first recurrent *Clostridiodes difficile* (*C. difficile*) infection. A barrier to increasing Fidaxomicin usage has been the medication’s potential high co-pay. The objective of this study was to evaluate the discharge success rate and financial/co-pay barriers for inpatients initiated on Fidaxomicin at our institution.

**Methods:**

Our cohort consisted of inpatients that received Fidaxomicin for the treatment of *C. difficile* during 2022. For inpatient discharges, a transitions of care (TOC) pharmacy evaluation is available for high co-pay medications. Charts were retrospectively reviewed for demographic, clinical, insurance, and pharmacy data.  The primary outcome was the proportion of patients successfully discharged on Fidaxomicin. Secondary outcomes included proportion of patients with co-pays >$50 and direct out-of-pocket patient cost after TOC pharmacy intervention.

**Results:**

29 patients were initiated on Fidaxomicin for CDI treatment in 2022. The majority of patients had Medicare (62%), a prior history of CDI (75%), were immunocompromised (55%), and met non-severe IDSA criteria (62%) (Table 1). 7% (2/29) switched to oral vancomycin early due to ID consult or stewardship recommendations and 28% (8/29) completed the course prior to discharge (Figure). All the remaining patients (19/19) discharged to extended care or home were able to be discharged on Fidaxomicin.

74% (14/19) of patients discharged on Fidaxomicin had TOC pharmacy evaluations with co-pay data available (Figure). 50% of these patients (7/14) had high co-pays (Figure) with a mean co-pay of $470.25 (range $65-1384.95) (Table 2). The majority (5/7) of the “high co-pay” group had Medicare; three Medicare patients had out-of-pocket cost reduced after TOC pharmacy intervention (Table 2). Post-TOC intervention, the mean patient out-of-pocket cost was $181.20 (range $0-566.44).

**Table 1:**
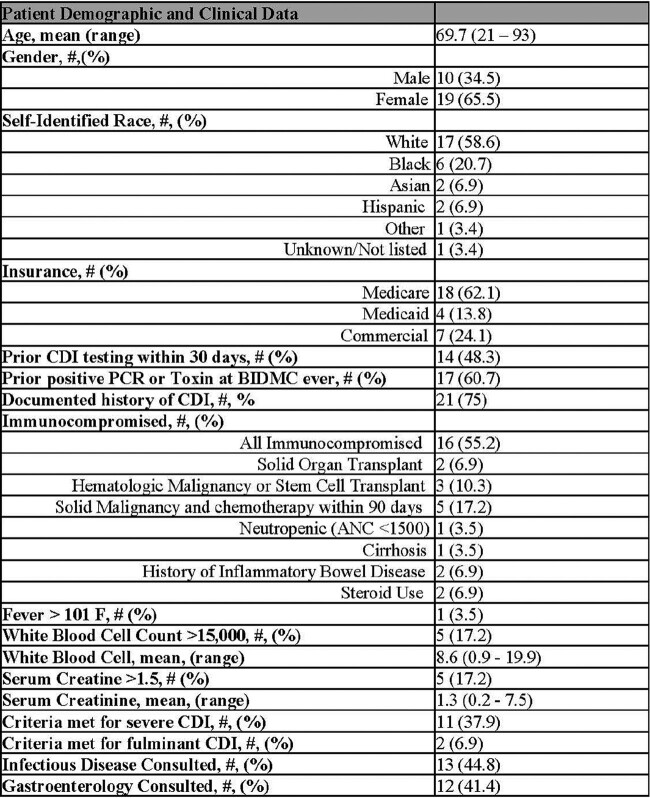
Patient Demographic and Clinical Data

**Table 2:**
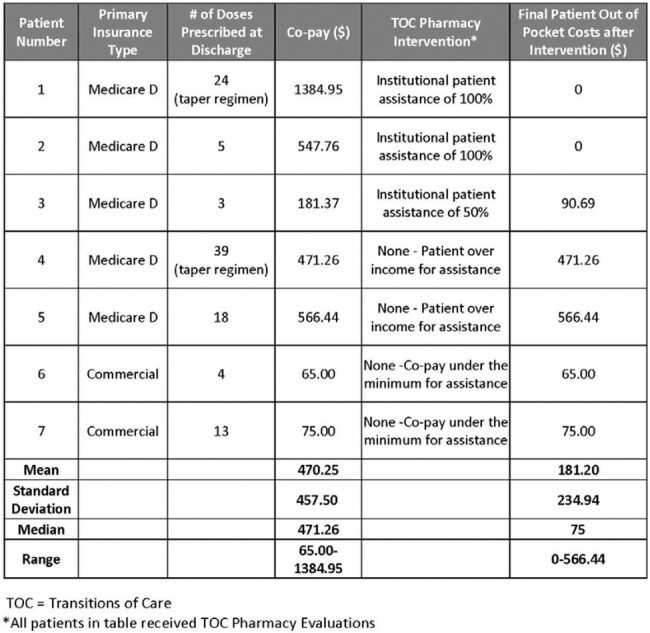
“High Co-Pay” Patient Group (defined as Co-Pay > $50)

**Figure:**
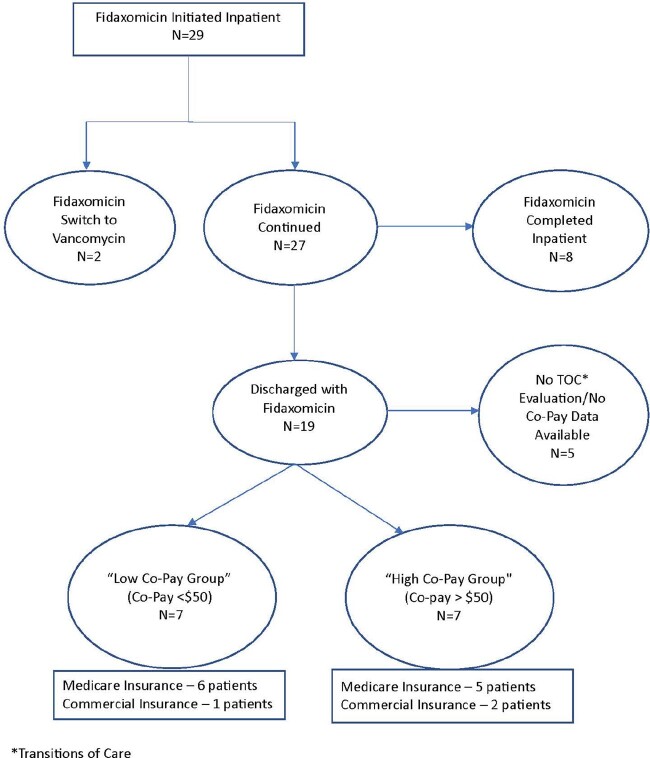
Fidaxomicin Prescribing Cascade

**Conclusion:**

All inpatients continued on Fidaxomicin for the treatment of *C. difficile* were able to be “successfully” discharged with the medication. However, the co-pay remains potentially high for certain individuals, particularly those with Medicare and prolonged tapers. TOC pharmacy evaluations, if available, are critical for financial assistance prior to discharge.

**Disclosures:**

**All Authors**: No reported disclosures

